# Delays in treating endometrial cancer in the South West of England

**DOI:** 10.1038/bjc.2011.173

**Published:** 2011-05-24

**Authors:** N Johnson, T Miles, D Bailey, K Tylko-Hill, N Das, G Ahson, K Waring, N Acheson, M Voss, J Gordon, S Keates-Porter, G Hughes, S Golby, E Fort, L Newton, V Nallaswamy, J Murdoch, R Anderson

**Affiliations:** 1 Gynaecology, PAW, Royal United Hospital Bath NHS Trust, Bath, UK; 2 CNS, Royal United Hospital Bath NHS Trust, Bath, UK; 3 South West Public Health Observatory, Grosvenor House, 149 Whiteladies Road, Bristol, UK; 4 Macmillan CancerVOICE, Bath, UK; 5 Royal Cornwall Hospitals NHS Trust, Truro, UK; 6 Royal Bournemouth & Christchurch Hospitals NHS Foundation Trust and Poole Hospital NHS Foundation Trust, Poole, UK; 7 University Hospital Bristol NHS Foundation Trust, Bristol, UK; 8 Royal Devon and Exeter NHS Foundation Trust, Exeter, UK; 9 Gloucestershire Hospitals NHS Foundation Trust, Cheltenham, UK; 10 CNS, Northern Devon Healthcare NHS Trust, Barnstaple, UK; 11 CNS, Weston Area Health NHS Trust, Weston Super Mare, UK; 12 Plymouth Hospitals NHS Trust, Plymouth, UK; 13 CNS, Taunton and Somerset NHS Foundation Trust, Taunton, UK; 14 CNS Yeovil District Hospital NHS Foundation Trust, Yeovil, UK; 15 CNS, Dorset County Hospital NHS Foundation Trust, Dorchester, UK; 16 South Devon Healthcare NHS Foundation Trust, Torbay, UK; 17 South West Regional Gynaecological Tumour Panel, Bristol, UK

**Keywords:** early diagnosis, cancer awareness, avoidable deaths, delay, early presentation, endometrial cancer

## Abstract

**Background:**

Poor cancer survival rates in the United Kingdom are often blamed on delayed medical care. A local audit of endometrial cancer revealed a variety of preventable delays. We surveyed practice in the South West of England to see if this was an isolated or widespread problem.

**Methods:**

All 15 hospitals in the South West of England collected information prospectively from all women with endometrial cancer over 3 months in the spring of 2009.

**Results:**

There were delays in all stages of the uterine cancer pathway. Excluding extraneous cases, 52% of women waited more than a month and 12% waited more than 6 months to see their GP from the onset of symptoms. Almost half the cases said they were unaware that abnormal bleeding was a symptom of cancer. Only a quarter of women had treatment within 31 days from the outpatient visit to first definitive treatment and 18% waited more than the target of 62 days for their treatment.

**Conclusions:**

Significant treatment delays occur because women do not report bleeding. If this is replicated throughout Britain, approximately 1000 women per year will delay presentation for at least 3 months and 600 will wait for more than 6 months.

Poor cancer survival rates in the United Kingdom (Sant *et al*, 2003, 2009; Berrino *et al*, 2007) are often blamed on diagnostic and treatment delays (Coleman *et al*, 2011). It has been suggested that delays might contribute to some of the differences in outcomes between rich and poor (Coleman *et al*, 2001) and black and minority ethnic populations and Caucasians (Jack *et al*, 2009). It could also account for the differences in survival observed between the United Kingdom and other comparable western European countries. The supporting evidence is complex, occasionally contradictory and is still incomplete. However, the overwhelming belief is that outcomes from cancer will be improved if delays in diagnoses can be avoided (Richards, 2009a) with the potential (based on data obtained 10 years ago) to avoid 500 deaths per year from uterine cancer (Abdel-Rahman *et al*, 2009). The National Awareness and Early Diagnosis Initiative (NAEDI) program is one of the key commitments of the British Government's Cancer Reform Strategy (Department of Health, 2007). The NAEDI pathway (Richards, 2009b) aims to improve cancer outcomes by examining the steps that contribute to a late diagnosis. The first step in the pathway focuses on the low public awareness of the signs and symptoms of cancer combined with negative beliefs about cancer and the perceived or actual barriers to accessing primary care services. The second step involves delays within primary care, usually due to failure to consider cancer as the diagnosis given all the potential competing benign causes. Delays in secondary care are usually due to system failures. This model encourages local care providers to eliminate significant delays at each of these stages. However, the first problem is to identify where any delays occur. The best way to answer this is to survey practice.

Our survey of practice began because a Macmillan CancerVOICE patient advocate identified several women whose survival chances from endometrial cancer were probably reduced because of a long duration between first symptoms and treatment. A subsequent retrospective audit of endometrial cancers between 2007 and 2008 in Bath, England confirmed that many women had not been referred through the two-week wait system and some women suffered significant delays due to poor triaging. A subsequent detailed medical chart review found that some women had their definitive and curative surgery within a week from the first symptom but others waited up to a year before a referral. Delays were due to poor patient knowledge, general practitioner's failure to refer or investigate cases despite a clear history of abnormal bleeding and the poor failsafe systems in the general gynaecological service. The first might be addressed by funding a public health campaign, the second might be addressed by better feedback and education to general practitioners and the third was addressed by establishing failsafe systems within the hospital unit. The South West (England) Gynaecological Oncology Group was concerned to know if this was a small isolated problem unique to one hospital catchment area or a far-reaching problem throughout the region. To see if the delays in uterine cancer diagnosis were endemic in England, the South West Gynaecology Tumour Panel (http://www.swpho.nhs.uk) agreed to survey the steps in the NAEDI pathway prospectively over 3 months.

## Materials and methods

This enquiry began after a hospital audit highlighted the time taken to treat some cases of endometrial cancer. There was a general agreement at a meeting of the South West (UK) gynaecological cancer tumour panel that there were other memorable cases of delayed diagnoses in women with endometrial cancer and that the delay might possibly have contributed to additional treatment and a poorer prognosis. This prompted a prospective observational study of all cases of endometrial cancer treated in the South West of England between 1 March 2009 and 31 May 2009. Cases were identified by theatre lists, local databases, pathology departments, coding departments and multidisciplinary meeting records to ensure capture of all cases. Data were collected by each participating hospital on data sheets. It focused on when women had their first bleed, reasons for any delay in diagnosis and the number of times women consulted their GP before endometrial sampling. This included a questionnaire to collect the experiences of women once they had been diagnosed with endometrial cancer. The data were sent centrally to the South West Cancer Intelligence Service (now the South West Public Health Observatory) for analysis and reporting. The primary audit target was set at 96 and 91% for secondary and tertiary care referrals, respectively, for definitive treatment to begin within 62 days from the GP's referral. It was felt that a survey of practice should have an accuracy of <±5%. This meant that we required a sample size of approximately 150 cases of endometrial cancer. This would have been sufficient to detect a rate of delayed diagnoses with an accuracy of ±4%, assuming negligible misreporting. Increasing the sample size would give minimal improvement and it was estimated that the project should take 3 months of prospective reporting based on an annual average of 630 new cases in the South West using data from 2002 to 2006. All hospitals that managed endometrial cancer in 2009 in the four Cancer Networks in the South West of England agreed to participate. The cancer networks comprised of the Avon Somerset and Wiltshire, Dorset, Peninsula and Three Counties Network. The NHS hospitals comprised of Dorset County Hospital NHS Foundation Trust, Gloucestershire Hospitals NHS Foundation Trust, North Bristol NHS Trust, Northern Devon Healthcare NHS Trust, Plymouth Hospitals NHS Trust, Poole Hospital NHS Foundation Trust, Royal Bournemouth & Christchurch Hospitals NHS Foundation Trust, Royal Cornwall Hospitals NHS Trust, Royal Devon and Exeter NHS Foundation Trust, Royal United Hospital Bath NHS Trust, South Devon Healthcare NHS Foundation Trust, Taunton and Somerset NHS Foundation Trust, University Hospitals Bristol NHS Foundation Trust, Weston Area Health NHS Trust and Yeovil District Hospital NHS Foundation Trust.

## Results

All fifteen NHS hospital trusts in the four cancer networks participated in the study. In all, 142 cases were reported (Table 1). The age distribution of endometrial cancer was typical of the disease. The average age was 68 (median 68; range 21–96). About a quarter were under the age of 60 and a quarter were over the age of 75. Nineteen women with endometrial cancer did not present with typical symptoms of abnormal bleeding to the GP. Some were direct referrals from urologists or A&E with pain or haemorrhage, and some had signs of metastatic disease at presentation and others had an abnormal ultrasound scan but no symptoms. This left 123 women with endometrial cancer confined to the uterus and cervix who presented with abnormal vaginal bleeding.

Half waited more than a month to see their GP after their first vaginal bleed. In all, 22% waited more than 3 months to see their GP and 12% waited more than 6 months before they went to see their GP. Some women approached their GP more than once. We could not tell how many times 5 of the 123 women saw their GP, leaving us with data on 118 women. Eighteen percent of these women with abnormal bleeding needed to visit their GP more than once before an investigation and one woman had to approach her GP 4–5 times after being told that her persistent post-menopausal bleeding was attributed to atrophy. The hospital appointments system provided 21% of the 123 women with an appointment within a week of the GP sending the referral letter. Thirty percent waited more than 2 weeks and 13% waited over a month.

Some women were able to give reasons for these delays. For example, 85 commented on their abnormal uterine bleeding and approximately half (*n*=41) said that they had no idea that it was a sign of possible cancer. Ten women (out of 72 responders) admitted that they delayed approaching their GP because they were scared about the possibility of cancer. Three out of 75 delayed seeking help because they felt that it had been difficult to access the GP, either because their own GP was on holiday or because full time work coincided with the surgery opening hours.

Eleven out of 83 responders reported that their GP did not make an urgent referral to the rapid access gynaecological services because their GP had told them that their bleeding was normal. One woman was told to watch and wait for a month before being referred. Seven out of 79 responders had a delay and they thought that this was because their GP did not use a two-week wait service. In fact, a review of the case notes showed that about a third of the women were not referred through the two-week wait system. Fifty five percent of those referred using the general referral system waited more than 2 weeks and a quarter waited over a month to be seen. In contrast, 89% of women were seen within 2 weeks if the cancer referral two-week wait referral system had been used (*χ*^2^=24.5518; df=3; *P*<0.0001), but that still meant that 11% waited more than 2 weeks to be seen. Four percent waited a month or more.

Once a woman had seen her GP, the next step in the NAEDI pathway was to have an endometrial biopsy, diagnosis and treatment. Of the 142 cases, 24 cases do not contribute to this part of the analysis because they were either emergency admissions, asymptomatic and diagnosed because a coincidental ultrasound was abnormal, or presented with symptoms of advanced disease. Only a quarter (30 of the 118) women had their first definitive treatment, usually hysterectomy within 31 days from the outpatient visit. Eighteen percent waited more than the target of 62 days for their treatment. Not all cases were straightforward. For example, one woman needed three biopsies before a diagnosis was made. One surgeon saw and diagnosed a cancer within a week but was told by hospital management that the target was 62 days and the cancer surgery had to be delayed so that the operating time could be devoted to sterilisation procedures because these women risked breeching the national 19-week waiting target. The waiting time for treatment was similar irrespective of the route of referral. Eighty percent of women referred using the suspected cancer or two-week wait criteria were treated within 2 months.

## Discussion

Some women with endometrial cancer in the South West of England experienced delays in their cancer journey due to poor health education, inefficient primary care referral practices and delays in initiating hospital treatment. The most significant observations are that about half of the women with cancer claimed to have no idea that abnormal vaginal bleeding could represent cancer, half took more than a month to see their GP from the onset of symptoms, a third of GPs failed to triage cases of abnormal bleeding to a cancer service and 18% of women waited longer than the target of 62 days for their treatment. Delays were due to multifactorial issues involving all aspects of the NAEDI pathway.

The delays in treatment at the hospital level were often for good reason and the vast majority of women are unlikely to have had their outcome affected adversely. The poor triaging by some GPs despite classic symptoms caused statistically significant delays. The occasional GP who failed to refer or investigate a woman as soon as she presented will, in retrospect be embarrassed, but a single example throughout the South West of the country probably represents a single clinical error rather than a system failure. It is delays caused by patients that are likely to cause the most important contribution to a potentially dangerous long symptom to treatment interval. The key to improving outcomes might be to focus resources on the one in eight women who waited more than 6 months before they went to see their GP. Ignorance of important symptoms and reluctance to attend the doctor are long-standing problems with the British population. Numerous surveys show that the British public are poorly educated in health issues and cancer risk symptoms (Wardle *et al*, 2001) and are inhibited by barriers to help seeking such as difficulty making an appointment, worry about wasting the doctor's time and worry about what would be found (Robb *et al*, 2009). There are numerous suggested strategies for improving public knowledge and tools to evaluate these interventions (Stubbings *et al*, 2009) but a systematic review of the world literature (Austoker *et al*, 2009) shows that there is very little evidence that interventions are effective at promoting earlier presentation. Exceptions are breast cancer (Catalano *et al*, 2003; Gabram *et al*, 2008), malignant melanoma (Rossi *et al*, 2000; MacKie *et al*, 2003), retinoblastoma (Leander *et al*, 2007) and tobacco use. However, the absence of evidence of an effect in uterine cancer is not evidence of ineffectiveness. Public information strategies probably work in breast cancer and melanoma care and this suggests that a greater public awareness about the seriousness of postmenopausal bleeding might be effective. An example of an intensive education campaign is the Eve Appeal (www.eveappeal.org.uk). This may lead to greater cancer awareness and earlier presentation over the short term but the effect may not be sustained and we do not know how to design a campaign to make it effective.

This is the first British survey we could find that focuses on endometrial cancer and other regions will need to see if the results are mirrored elsewhere. The strength of the study is that it reflects current practice throughout a large region, all women were captured and it was prospective. This minimises sampling bias, reduces recall bias and the high ascertainment rate implies that the results will be internally valid. External validity is more difficult to establish because of the lack of other similar work but a Danish survey of all gynaecological cancers also highlighted diagnostic delays in all parts of the diagnostic pathway (Robinson *et al*, 2009). Although the UK has national targets and uniform referral health care systems, there will be regional variations because attitudes, education and resources vary. The data will not be timeless and will not reflect care outside the NHS. Another problem is that we do not know how to respond to our findings. On the face of it, this study suggests that a campaign to get GPs to use the fast track cancer referral pathway and a public health campaign might save lives. A greater use of the fast track system might swamp resources, dilute expertise and delay the diagnosis in higher risk women. Finally, we don’t know if the prompt diagnosis of uterine cancer is important. With the exception of breast cancer (Richards *et al*, 1999), the linkage between delay and poor survival has been difficult to prove from observational studies. Trial data in this area are lacking and observational studies often show no association or negative ones. Indeed a survey in Scotland reported the apparently paradoxical finding that patients with longer delays may have better survival rates (Crawford *et al*, 2002). How might this be explained? To quote Mike Richards, National Clinical Director for Cancer, most of the reported studies fail to report the nature of the first symptom and patients with the most sinister symptoms in terms of prognosis may present rapidly to health services, while those with other symptoms may still have early-stage disease even after several months. The logical explanation for this is that doctors fast track patients with more obvious and advanced cancers (Neal, 2009). This paradox is not unique to cancer care (Turner and Al-Chalabi, 2002). Therefore, it seems sensible to assume that a 6 months delay is dangerous. If this data were reflected across the country, approximately 1000 women per year delay presenting to their GP by at least 3 months and 600 wait over 6 months. We have invested enormous amounts of finance into improving cancer pathways. More investment is likely to suffer from diminishing returns. Perhaps it is time to invest in public education.

## Figures and Tables

**Table 1:** Time to pass through the stages in the NAEDI pathway

	**Number of cases (%)**
*Time from first symptom to first GP visit*	
Up to 1 month	60 (49)
1–2 months	21 (17)
2–3 months	15 (12)
3–6 months	12 (10)
>6 months	15 (12)
Presented with symptoms other than abnormal bleeding	19
	
*Time from GP referral to first outpatient visit*	
0–7 days	26 (21)
8–14 days	60 (49)
15–31 days	22 (18)
>31 days	15 (12)
N/A	9
Data not complete or non-verifiable	10
	
*Time from first outpatient visit to date of first treatment (e.g., hysterectomy)*	
0–31 days	30 (25)
32–62 days	67 (57)
>62 days	21 (18)
Not presenting through an outpatient clinic	15
NK	9

Abbreviations: NAEDI=National Awareness and Early Diagnosis Initiative; NA=Not known.
